# A Case of* Mycobacterium riyadhense* in an Acquired Immune Deficiency Syndrome (AIDS) Patient with a Suspected Paradoxical Response to Antituberculosis Therapy

**DOI:** 10.1155/2016/5908096

**Published:** 2016-09-14

**Authors:** Maged Omar Al-Ammari, Samar Assem Badreddine, Hani Almoallim

**Affiliations:** ^1^Department of Internal Medicine, Dr. Soliman Fakeeh Hospital, Jeddah, Saudi Arabia; ^2^Department of Medicine, Medical College, Umm Alqura University, Makkah, Saudi Arabia

## Abstract

A 30-year-old male patient with acquired immune deficiency syndrome (AIDS) on highly active antiretroviral therapy (HAART) presented with clinical picture suggestive of pulmonary tuberculosis. He was commenced on antituberculosis therapy (ATT) with signs of improvement. Then he developed cervical lymph node abscess which was drained. Steroid was started for presumed paradoxical response to ATT which results in clinical regression. The culture result revealed* Mycobacterium riyadhense*. This report addresses the rarity of this bacteria in medical literature. It reviews clinical presentations and medical treatment particularly in the setting of coinfections.

## 1. Introduction

*Mycobacterium riyadhense* is a slowly growing, nonchromogenic nontuberculous mycobacterium with unique biochemical properties and with mycolic acid profile that support separate species status. It produces rough, white colonies after 28 days of incubation at 36°, growth is slower at 25°–30°, and no growth occurs at 42°. It got its name (Riyadhense) after Riyadh, capital of the Kingdom of Saudi Arabia and origin of the patient from whom the first strain of this organism was isolated [1]. Pulmonary tuberculosis is one of the acquired immune deficiency syndrome (AIDS) defining illness [2]. Nontuberculous mycobacteria (NTM) has been reported in human immunodeficiency virus infection [3]. We are here reporting a rare mycobacterium (*Mycobacterium riyadhense*) in the setting of a patient with AIDS on highly active antiretroviral therapy (HAART).

## 2. Case Report

A 30-year-old male was diagnosed with acquired immune deficiency syndrome (AIDS) at age of 28 years and presented to Dr. Soliman Fakeeh Hospital (DSFH) with history of fever, cough. and weight loss of 3-week duration. His past medical history was positive for treated* Pneumocystis jiroveci* pneumonia. Home medications were efavirenz, emtricitabine, tenofovir, and trimethoprim/sulfa for secondary prevention of* Pneumocystis*. On admission his chest X-ray showed multiple well defined cavitary lesions seen within the right upper lobe and his sputum was positive for acid fast bacilli resembling* Mycobacterium tuberculosis*. Sputum was not sent for tuberculosis culture. His CD4 on admission was 724 cell per cubic millimeter compared to 160 cells per cubic millimeter before starting HAART, and his viral load on admission was 1350 copies/mL compared to 292000 copies/mL before starting HAART. The patient diagnosis was presumed to be pulmonary tuberculosis and he was started empirically on isoniazid, rifampicin, ethambutol, and pyrazinamide. Within a couple of weeks after starting antituberculosis therapy (ATT) he developed rifampicin induced hepatotoxicity and it was replaced with moxifloxacin. The patient improved clinically on the new ATT. Around 12 weeks after being on ATT, he developed a large, tender, and erythematous right neck swelling. There were no associated systemic manifestations of fever or weight loss. Ultrasound examination of the swollen part of the neck showed necrotic lymph nodes with abscess formation. Diagnosis of paradoxical inflammatory syndrome was suspected. The patient was maintained on the same ATT and started on a course of tapering dose of steroids. Significant response of the neck swelling to steroid was noted in 2 months' time.

The neck swelling recurred with tenderness after 2 weeks of completing the course of steroid and reached the same size as it was before starting steroids. He did not have any associated systemic manifestations and was otherwise clinically improving, which made the possibility of multi-drug-resistant tuberculosis (MDR-TB) unlikely, but not completely excluded. Around 60 cc of purulent material was aspirated from the swelling and was sent for mycobacterial culture. He was restarted on steroids and was continued on the same ATT. Again, a dramatic improvement of the neck swelling and tenderness occurred few days after starting the steroids. The pus culture came back positive for* Mycobacterium riyadhense*. He continued the same ATT. He completed one month course of tapering dose of steroids. The neck and lung infections continued to improve without any need for any additional courses of steroids.

## 3. Discussion

The Middle-East region is an endemic area for* Mycobacterium tuberculosis*, with incidence by WHO estimated to be 14 per 100000 population in Saudi Arabia and 18 per 100000 population in Bahrain [4]. The patient in our report is living in Jeddah in the western region of Saudi Arabia. The previously reported cases of* Mycobacterium riyadhense* involved cases from Riyadh and Jeddah in Saudi Arabia, Bahrain, France, and Korea [1, 5–8]. That makes 6 out the 8 reported cases come from an endemic area for* Mycobacterium tuberculosis*.

Clinically there was a pulmonary and cervical lymph nodes involvement in the reported patient. Four of the previously reported cases [5, 6, 8] had pulmonary involvement, two from Riyadh had only bony involvement (maxillary sinus and spine) [1, 7], and a case from Jeddah had brain with bony involvement [7] as shown in Table 1. These findings suggest that this strain, just like* Mycobacterium tuberculosis*, can cause both pulmonary and extra pulmonary manifestations.

The patient clinically improved on antituberculosis therapy (ATT) which included isoniazid, rifampicin, pyrazinamide, and ethambutol, as did all the previously reported cases [1, 5–8]. In one of these cases [6] it was noted that even though in vitro susceptibility for the involved strain showed sensitivity to ciprofloxacin and clarithromycin, the patient relapsed on a combination of both and only responded to standard ATT. In another case [5] it was resistant to isoniazid but still showed improvement on standard ATT, as shown in Table 1. These findings as in the case reported here show the similarities in management of* Mycobacterium tuberculosis* and* Mycobacterium riyadhense*.

Despite these similarities, the diagnosis of* Mycobacterium riyadhense* was difficult to establish in this case. The entire clinical course could have been attributed to* Mycobacterium tuberculosis* only. However, the presence of strongly positive culture for* Mycobacterium riyadhense* from the pus obtained from the lymph node argues against this. There is a remote possibility given the lack of the culture of the sputum of coinfection with two different mycobacterial organisms. All of this is further to emphasize the need for cultures to establish the diagnosis.

The development of paradoxical clinical worsening while on proper therapy is a phenomenon called paradoxical inflammatory syndromes that is well known with tuberculosis [9] and is known to develop 6–8 weeks after starting patients on proper ATT. It consists of worsening clinical condition after initial response and usually manifests as development of new swelling of lymph nodes, lung infiltrates, pleural effusions, or central nervous system tuberculosis. It responds to courses of steroids [10]. The diagnosis of this paradoxical phenomenon can only be ascertained when other differential diagnoses such as secondary infections, inadequate antituberculosis therapy as a result of drug resistance, poor compliance, and adverse reactions due to therapy are all excluded. The culture of the neck abscess in the case presented grew* Mycobacterium riyadhense* that responded well to the standard ATT clinically. It is not clear if the lung involvement was due to* Mycobacterium tuberculosis* or* Mycobacterium riyadhense*, as there were no mycobacterial cultures sent from his sputum, despite the fact that there was definitive lymph nodes involvement with* Mycobacterium riyadhense*. There is a possibility that the paradoxical response to treatment can occur with* Mycobacterium riyadhense* just like* Mycobacterium tuberculosis*. This is possibly based on the observation that the lymph nodes involvement developed after good initial response to ATT. In addition, the patient showed significant improvement on steroids. This can be another aspect of similarity in clinical presentation of* Mycobacterium riyadhense* with* Mycobacterium tuberculosis* that was not reported in the previous cases [1, 5–8].

The patient was on highly active antiretroviral therapy (HAART) for the last 2 years with a high viral load and CD4. The relationship to the development of the paradoxical reaction to the high CD4 count and the viral load at the time of the presentation is an area of further research. There was no association found between development of paradoxical reaction and baseline CD4 count or the viral load in HIV-positive patients with tuberculosis treatment [11].

Absence of contact history does not completely exclude the respiratory/airborne mode of transmission. The patient denied any history of contact with patients that have respiratory symptoms or lung infections. Similar lack of exposure history was elicited in previously reported cases [1, 5–8]. Whether mode of transmission of* Mycobacterium riyadhense* is the same as that of* Mycobacterium tuberculosis* or not still needs to be clarified.

*Mycobacterium riyadhense* could be more prevalent than what is known. It is a common practice in Saudi Arabia to empirically treat patients with lung infiltrates and positive sputum for acid fast bacilli with ATT without performing mycobacterial culture. Probably, few labs are prepared to perform mycobacterial culture. The same practice has been followed in the reported case. The message that should be conveyed here is to consider mycobacterial culture in every suspected patient with tuberculosis regardless of any other findings.

The Middle-East region is harboring most of the* Mycobacterium riyadhense* cases until now. It can present with pulmonary, brain, lymph node, or bone manifestations. It showed clinical improvement on standard ATT. The paradoxical inflammatory syndromes can be observed with* Mycobacterium riyadhense*. The mode of transmission still needs to be elucidated. Overall it is a rare disease and should be addressed in cases of tuberculosis with proper cultures.

## Figures and Tables

**Table 1:** The different clinical characteristics in the previous reported cases in comparison to the reported patient.

Case	Age (years), gender	Country	Sites of involvement	Initial antituberculosis therapy	HIV
van Ingen et al. [1]	19, M	Riyadh	Bone, maxillary sinus	INH, RIF, EMB	Not reported
Godreuil et al. [6]	43, M	Bahrain	Lung	CLR, CIP	Not reported
Godreuil et al. [6]	39, F	France	Lung	INH, RIF, EMB, PZA	Not reported
Choi et al. [5]	38, F	Korea	Lung	INH, RIF, EMB, PZA	Not reported
Saad et al. [7]	18, F	Jeddah	Brain and skull frontal bone	INH, RIF, EMB, PZA	Not reported
Saad et al. [7]	24, F	Riyadh	Spine	INH, RIF, EMB, PZA	Not reported
Garbati and Hakawi [8]	54, M	Riyadh	Lung	INH, RIF, EMB, PZA	Positive
Reported case	30, M	Jeddah	Lymph node and lung	INH, RIF^*∗*^, EMB, PZA	Positive

M, male; F, female; INH, isoniazid; RIF, rifampicin; EMB, ethambutol; CLR, clarithromycin; CIP, ciprofloxacin; PZA, pyrazinamide.

^*∗*^Rifampicin was changed to moxifloxacin few weeks later due to drug induced hepatotoxicity (DIH).
